# Clinically Translatable Mutation‐Based Biomarkers in Ascending Aortic Aneurysm: A Bibliometric Study

**DOI:** 10.1155/humu/3155191

**Published:** 2026-05-25

**Authors:** Fan Yang, Tan Yang, Xiaojun Xie, Qi Yang, Fang Wang

**Affiliations:** ^1^ Department of Cardiovascular Surgery, The Affiliated Hospital, Southwest Medical University, Metabolic Vascular Diseases Key Laboratory of Sichuan Province, Key Laboratory of Cardiovascular Remodeling and Dysfunction, Luzhou, Sichuan, China, utsouthwestern.edu

**Keywords:** ascending aortic aneurysm, bibliometrics, genetic variant, heritable thoracic aortic disease, pathogenic variant, risk stratification

## Abstract

Ascending aortic aneurysm is often clinically silent until rupture or acute dissection, and reliance on diameter‐based thresholds leaves substantial risk heterogeneity that may be reduced by genetic variant–informed biomarkers. In plain language, clinically translatable variant biomarkers are genetic findings that can change diagnosis, family screening, surveillance intensity, or prophylactic surgical timing. We performed a cross‐database bibliometric study to delineate the intellectual structure and thematic evolution of clinically translatable variant‐based biomarker research in ascending aortic aneurysm. Web of Science Core Collection, Scopus, and PubMed were queried using concept‐driven terms spanning thoracic/ascending aneurysm phenotypes, genomic variation, and biomarker/clinical translation; searches and exports were executed on January 10, 2026, for publications from 1997 to 2025. After harmonization, deduplication, and adjudicated screening of titles/abstracts and metadata, 1346 eligible journal articles and reviews were analyzed using bibliometrix and VOSviewer to compute productivity/impact indicators and construct co‐authorship, co‐citation, and keyword co‐occurrence networks with temporal overlays. The literature exhibited rapid growth (15.35% annual growth rate) across 478 sources, with a mean of 8.94 co‐authors per document, 24.67% international co‐authorship, an average article age of 9.28 years, and 30.68 citations per article. The United States was the dominant hub (405 articles and 17,383 citations), with a densely connected Western European cluster and accelerating output from China (155 articles) but lower citation intensity. Conceptual cores were anchored in syndromic and heritable thoracic aortic disease genetics (e.g., Marfan and Loeys–Dietz syndromes; FBN1 and TGF‐beta signaling), increasingly linked to diagnosis, variant interpretation, risk stratification, guidelines, and management. Life cycle modeling suggested an approach to publication peak around 2028, implying a shift from gene discovery toward variant interpretation, implementation, and equity‐conscious validation. Notably, peripheral participation from low‐resource regions remained sparse overall.

## 1. Introduction

Ascending aortic aneurysm is a clinically consequential aortopathy that can remain silent until catastrophic rupture or acute dissection, making timely risk stratification and surveillance central to prevention [1–3]. Current clinical decision‐making still relies heavily on anatomic criteria, particularly aortic diameter and growth rate, yet adverse events may occur outside conventional thresholds, and patients with similar imaging phenotypes can experience markedly different trajectories [4, 5]. In this manuscript, clinical risk stratification refers to estimating who is more likely to experience rapid enlargement, dissection at smaller diameters, pregnancy‐related complications, or earlier need for prophylactic surgery so that imaging intervals, family screening, referral intensity, and operative timing can be individualized [2, 4, 5]. In parallel, the last three decades have clarified that a substantial proportion of ascending aortic aneurysm is genetically mediated, spanning syndromic entities and nonsyndromic heritable thoracic aortic disease, as well as genetically influenced forms linked to bicuspid aortic valve and related aortopathies [6, 7]. Within this landscape, genetic information has moved beyond etiologic labeling and increasingly functions as a clinically actionable signal: Pathogenic or likely pathogenic variants can refine diagnosis, identify high‐risk families, prompt cascade screening, and influence surveillance intensity and surgical timing, particularly when genotype–phenotype correlations and outcome data support earlier intervention for specific genotypes.

Although we retain the commonly indexed phrase “mutation‐based biomarkers” in the title to mirror the source literature, we use “genetic variant” as the preferred umbrella term throughout the manuscript and reserve “pathogenic/likely pathogenic variant” for findings with established clinical actionability. In plain language, clinically translatable variant‐based biomarkers are genetic findings that can move from a sequencing report into real clinical actions, such as confirming diagnosis, prompting cascade screening, intensifying imaging surveillance, or informing prophylactic surgical timing. We chose the ascending aortic aneurysm as the primary unit of analysis because it is the segment in which genotype most directly intersects surveillance strategy and prophylactic surgery. Overlap with acute dissection, bicuspid aortic valve aortopathy, syndromic heritable thoracic aortic disease, and nonsyndromic familial cases was retained when studies substantively addressed ascending/thoracic aneurysm risk, diagnosis, or management, but not when they focused exclusively on unrelated vascular territories or nontranslational genetics [2, 6–8].

However, translating variant signals into deployable biomarkers for routine care remains uneven. Penetrance refers to the proportion of variant carriers who actually manifest disease, whereas variable expressivity refers to differences in age at presentation, aortic size, extra‐aortic features, and event severity among carriers of the same gene or variant [9–11]. Variants of uncertain significance are particularly challenging in aortopathy because they are common on multigene panels yet, in the absence of segregation, functional, or outcome evidence, should not by themselves trigger irreversible decisions such as earlier prophylactic surgery; at the same time, they complicate counseling and cascade testing in families with compelling phenotypes [9–11]. In this context, genotype–phenotype correlation means a reproducible relationship between a specific gene, variant class, or pathway and observed clinical features such as age at onset, growth rate, dissection at smaller diameters, extra‐aortic manifestations, or operative outcomes; such correlations are strengthened by family segregation, expert variant curation, functional assays, deep phenotyping, and longitudinal replication across independent cohorts [6, 8, 10]. As sequencing becomes increasingly accessible, the field has expanded from classic single‐gene paradigms toward multigene panels, pathway‐centric frameworks (notably extracellular matrix and TGF‐beta‐related signaling), and integrative strategies that attempt to connect genetic variants with imaging, pathology, and clinical endpoints [12, 13]. This expansion has also increased fragmentation: Evidence is distributed across genetics, cardiology, cardiovascular surgery, and rare disease outlets, while multicenter registries and guideline‐facing translational studies coexist with mechanistic and variant discovery work that does not always converge on clinical utility [14, 15]. As a result, it is difficult to appraise, at a field level, how research on variant‐based biomarkers in ascending aortic aneurysm has evolved, which intellectual lineages dominate translation, where collaboration is concentrated, and which topics represent sustained cores versus emerging opportunities.

In this context, bibliometric mapping offers a complementary lens to conventional narrative review. By integrating citation structure, co‐authorship patterns, and keyword dynamics, bibliometrics can reveal the field′s organizing hubs, the references and journals that anchor shared concepts, and the thematic pathways through which variant findings have been converted (or not yet converted) into clinically relevant biomarkers [16–18]. Such mapping is particularly useful for domains like ascending aortic aneurysm, where clinical translation requires coordinated accumulation of rare familial cases, standardized phenotyping, longitudinal follow‐up, and multidisciplinary expertise [8, 19, 20]. To make the analysis easier for clinical readers, we define specialized bibliometric terms in plain language at first use and interpret the maps in relation to concrete diagnostic, surveillance, and operative decisions. A bibliometric study can therefore help clarify how discovery‐driven genetics has interfaced with risk prediction and management, identify underconnected regions of the collaboration network, highlight topics that may be disproportionately influential relative to their publication volume, and reveal implementation gaps that matter to guideline development, research prioritization, and future collaboration.

We performed a cross‐database bibliometric study to delineate the knowledge structure and thematic evolution of clinically translatable variant‐based biomarkers in ascending aortic aneurysm over nearly three decades. Using an a priori protocol and harmonized records retrieved from major bibliographic databases, we quantified scientific production and impact, mapped collaboration across countries and institutions, and constructed networks of co‐citation and keyword co‐occurrence to characterize conceptual clusters and temporal shifts. By triangulating these perspectives with complementary science mapping tools, this study is aimed at providing an integrated field‐level account of how variant‐based biomarker research in ascending aortic aneurysm has matured and at informing clinical guideline development, research prioritization, collaboration/network building, and identification of implementation gaps.

## 2. Materials and Methods

### 2.1. Corpus Definition and Data Retrieval

We performed a cross‐database bibliometric study to map the intellectual and thematic structure of research on clinically translatable variant‐based biomarkers in ascending aortic aneurysm. Records were retrieved from Web of Science Core Collection, Scopus, and PubMed to balance citation‐rich indexing with broad biomedical coverage. To minimize time‐dependent variation in database content and citation accrual during data extraction, all searches and exports were executed on a single day (January 10, 2026) using an a priori protocol and a fixed publication window from January 1, 1997, to December 31, 2025. We chose 1997 as the lower bound not merely for convenience but because the late 1990s mark the beginning of the modern gene discovery and clinically interpretable sequencing era for heritable aortopathy literature, whereas earlier records were sparse and less comparable to the contemporary translational field [21–23]. The query logic was concept‐driven and built around three intersecting domains: ascending/thoracic aortic aneurysm phenotypes, genomic variation, and biomarker/clinical translation, combined with proximity operators (where supported) and database‐specific controlled vocabulary and field tags. Because clinically relevant literature often spans aneurysm, dissection, bicuspid aortic valve aortopathy, syndromic HTAD, and nonsyndromic familial disease, the search was intentionally permissive at retrieval and more specific at screening. For each database, we exported full bibliographic records with available abstracts, author keywords, indexed terms, affiliations, and cited references in formats compatible with downstream parsing (plain text and CSV for Web of Science and Scopus and MEDLINE/XML for PubMed). Where permitted by the platform, exports were performed without language restrictions at the query stage; language filtering, document‐type restrictions, and relevance adjudication were applied during subsequent screening to avoid premature exclusion of potentially eligible studies and to enforce consistent inclusion rules across databases.

### 2.2. Eligibility Criteria and Adjudicated Screening

We prespecified eligibility criteria to ensure that the final analytic corpus represented peer‐reviewed, citable contributions that could substantively inform variant‐linked biomarker translation in the context of ascending aortic aneurysm. Only journal articles and reviews were eligible; all other document types (e.g., but not limited to meeting abstracts, proceedings papers, editorials, letters, notes, corrections, book chapters, and early‐access items lacking final bibliographic completeness) were excluded. After importing all records into a unified screening workspace, the team conducted a two‐stage screening process. First, titles, abstracts, and available indexed terms were screened to remove records that were clearly outside scope (e.g., unrelated vascular territories, nonaortic aneurysm entities without ascending/thoracic relevance, or genetics/biomarker concepts detached from clinically oriented translation). Second, for records passing the initial screen, the full record metadata were reviewed to confirm that the study focus aligned with variant evidence linked to biomarker development, validation, risk stratification, diagnosis, prognosis, or clinically actionable surveillance pathways relevant to ascending aortic aneurysm. Screening was performed independently by two trained reviewers; disagreements were resolved through consensus discussion, with arbitration by a senior investigator when consensus was not reached. Operationally, a disagreement meant any discordant judgment about phenotype relevance, the degree of clinical translatability, whether genetic evidence was central rather than incidental, or whether document type/metadata completeness met eligibility. Consistency was maintained by applying the same prespecified eligibility rules to all records and by prioritizing substantive study focus over database‐assigned indexing terms. Throughout screening, we treated database‐indexed keywords and machine‐assigned terms as supportive rather than determinative, prioritizing the study′s substantive content as reflected in the title/abstract and, when necessary, journal context and indexing notes. The final corpus comprised 1346 eligible records within the prespecified time window, reflecting the application of these criteria after removal of duplicates and nonconforming formats and serving as the basis for all subsequent descriptive and network analyses.

### 2.3. Cross‐Database Harmonization, Deduplication, and Field Standardization

Because records were sourced from three databases with partially overlapping coverage and heterogeneous metadata conventions, we implemented a structured integration pipeline before analysis. All exports were parsed in R and normalized to a common schema encompassing document identifiers (DOI, PMID, and Scopus EID, where available), bibliographic descriptors (title, year, source, and volume/issue/pages), authorship (full names and initials), affiliations, countries, abstracts, author keywords, indexed terms, and cited references. Deduplication proceeded in a staged fashion: (i) deterministic matching on DOI/PMID, (ii) deterministic matching on exact title plus year, and (iii) probabilistic matching using normalized titles (case‐folding, punctuation removal, and whitespace normalization) combined with first author and source, followed by manual review of borderline pairs. When duplicates were detected, we retained a single master record while preserving the union of nonconflicting fields (e.g., augmenting missing abstracts or keywords from an alternative source) and keeping provenance flags to track which database(s) contributed each element. Author and institution names were standardized through rule‐based cleaning (e.g., unifying common abbreviation patterns, resolving inconsistent punctuation, and harmonizing diacritics) and through targeted disambiguation where high‐impact nodes showed clear fragmentation. Country assignments were derived from affiliation strings using a controlled mapping table to reconcile alternate spellings and geopolitical variants. For keyword analysis, we applied a thesaurus‐driven consolidation to merge lexical variants (singular/plural, hyphenation, and British/American spelling) and to reduce semantic duplication across synonymous expressions while preserving clinically meaningful distinctions. Merge rules were limited to terms whose clinical meaning was unchanged by standardization; nonmerge rules explicitly kept apart phenotypes, genes, and variant classes that carry different clinical implications (e.g., aneurysm vs. dissection, bicuspid aortic valve aortopathy vs. syndromic HTAD, FBN1 vs. TGFBR2, and pathogenic/likely pathogenic variants vs. variants of uncertain significance). Cited references were standardized (journal abbreviations, year, volume, and page) to improve the stability of co‐citation structures.

### 2.4. Bibliometric Indicators and Analytical Conventions

We summarized scientific production and impact using established bibliometric indicators calculated on the integrated corpus. Core descriptive outputs included annual publication counts, cumulative growth patterns, and citation‐based measures computed at the record level and aggregated by sources (journals), authors, institutions, and countries. Here, annual growth rate denotes the compounded year‐to‐year increase in publication output across the study period, average document age denotes the mean time from publication to the extraction date, citations per article denote the mean citation count per included record, and local citations denote citations received from within the analyzed corpus rather than from the wider database universe. Because citation counts differ by database and are time‐sensitive, all citation metrics were treated as a snapshot corresponding to the unified extraction date; for integrated records, citation values were preferentially drawn from citation‐indexed sources when available, with provenance retained to support sensitivity checks. We computed productivity and influence indicators such as total publications, total citations, citations per document, and author‐level indices (e.g., h‐type summaries where supported by available metadata), and we characterized collaboration using counts and proportions of single‐country publications (SCPs) versus multiple‐country publications (MCPs), as well as co‐authorship structures at author, institutional, and national levels. For country‐level reporting, we distinguished outputs by corresponding author affiliation to approximate the coordinating address or leadership locus of a paper in bibliometric mapping while recognizing that leadership can be distributed across first authors, senior authors, recruiting centers, sequencing cores, and steering committees in multicenter consortia. We summarized the balance between SCPs and MCPs as a proxy for internationalization. When comparing entities with different publication volumes, we emphasized paired reporting of productivity and impact to avoid conflating scale with influence, and we interpreted rankings in light of the underlying distributional skew typical of biomedical citation data and of citation advantages conferred by database coverage, English‐language visibility, publication age, and field‐specific citation practices [24–26]. Analyses were performed using VOSviewer and the bibliometrix R package (via scripted workflows to ensure reproducibility), with consistent handling of fractional versus full counting depending on the analytic goal: full counting for descriptive tallies (readability and alignment with common reporting) and fractional counting for selected collaboration networks to mitigate inflation from large consortia. All transformations, exclusions, and derived variables were applied identically across the integrated dataset to preserve internal consistency.

### 2.5. Statistical and Visualization Methods

To interrogate the field′s structure beyond descriptive indicators, we conducted complementary science mapping analyses using VOSviewer and bibliometrix, each applied to the same harmonized corpus but optimized for different inferential targets. In VOSviewer, we constructed networks for co‐authorship (authors, institutions, and countries), co‐occurrence (author keywords and, where appropriate, indexed terms), citation and bibliographic coupling (documents/sources), and co‐citation (cited references and cited sources). Conceptually, co‐citation captures earlier references that are cited together by later papers, whereas bibliographic coupling captures current papers that resemble one another because they cite the same earlier literature [16–18]. Networks were built with minimum‐occurrence thresholds selected to balance interpretability with coverage, and edge weights were defined using link strength as implemented in VOSviewer, where link strength refers to the cumulative weight of a node′s connections to other nodes, with association strength normalization to stabilize comparisons across nodes with heterogeneous activity. Community structure was detected using modularity‐based clustering, and we generated both cluster and overlay visualizations; overlays used average publication year to reveal temporal shifts in thematic emphasis, while density views were used to highlight concentrated conceptual cores versus peripheral or emerging topics. In bibliometrix, we replicated and extended these perspectives through three‐field relationships, thematic mapping (centrality–density frameworks), trend topic timelines, and collaboration maps that complement VOSviewer′s graph‐theoretic layout with interpretable summary statistics. Throughout, keyword cleaning and thesaurus rules were held constant between tools to ensure that thematic interpretations were attributable to underlying literature rather than preprocessing artifacts. The workflow and reporting were aligned with established bibliometric guidance to improve transparency and interpretability for clinical readers [16–18, 25].

## 3. Results

### 3.1. Analysis of the Overall Situation in This Field

**Figure 1:**
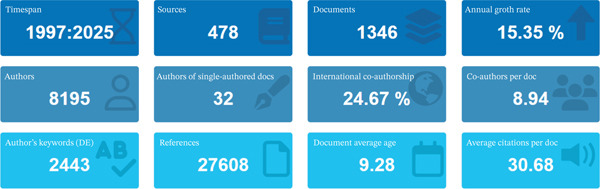
Overall bibliometric profile of research on clinically translatable variant‐based biomarkers in ascending aortic aneurysm (1997–2025).

**Figure 2:**
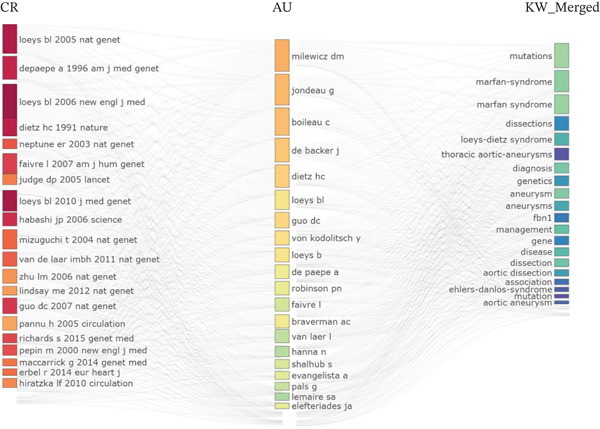
Three‐field plot linking influential cited references, leading authors, and high‐frequency keywords in the field.

From 1997 to 2025, research on clinically translatable variant‐based biomarkers in ascending aortic aneurysm has expanded from a marginal topic to a rapidly growing, collaboration‐intensive field, with 1346 documents published across 478 sources and an annual growth rate of 15.35% (Figure 1). The average document age of 9.28 years and 30.68 citations per article indicate a relatively mature but still active literature in which early foundational papers continue to shape current work. The very low number of single‐authored papers (*n* = 32) and a mean of 8.94 co‐authors per document, together with nearly one quarter of publications involving international co‐authorship (24.67%), show that progress in this area depends on multicenter consortia that can assemble sufficiently large pedigrees and patient cohorts; integrate cardiovascular genetics, imaging, and surgery; and share high‐throughput sequencing infrastructure. The three‐field map linking cited references, leading authors, and high‐frequency keywords (Figure 2) demonstrates that the intellectual core of the field is anchored in seminal gene discovery and syndrome definition studies (e.g., Loeys, Dietz, Neptune, and colleagues) that established FBN1 and TGF‐beta pathway variants and syndromic entities such as Marfan syndrome, Loeys–Dietz syndrome, and related connective tissue disorders as major substrates of thoracic aortic aneurysm and dissection. These highly cited references are tightly coupled to a network of contemporary clinical and translational leaders (e.g., Milewicz, Jondeau, Boileau, De Backer, Guo, Shalhub, and Evangelista), whose work links variant profiling to risk stratification, dissection prediction, and management decisions. In clinical terms, this risk stratification literature is about who should undergo closer imaging follow‐up, broader family screening, pregnancy counseling, or prophylactic surgery before standard size thresholds. On the thematic axis, dominant keywords such as “mutations,” “genetics,” “aortic aneurysm,” “aortic dissection,” “diagnosis,” and “management” underscore that the field has moved from descriptive syndrome reports toward variant‐informed classification and clinically actionable biomarkers. Figures 1 and 2 depict a research landscape characterized by sustained citation impact, strong networked collaboration, and a conceptual focus on heritable thoracic aortic disease, driven by the need to translate rare but high‐penetrance genetic information into concrete decisions about surveillance and prophylactic intervention.

### 3.2. Publication Volume Trends Over Time and Future Projections for This Field

**Figure 3:**
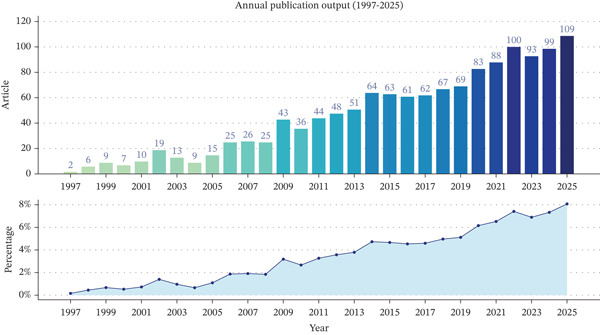
Annual number and percentage of publications on clinically translatable variant‐based biomarkers in ascending aortic aneurysm, 1997–2025.

**Figure 4:**
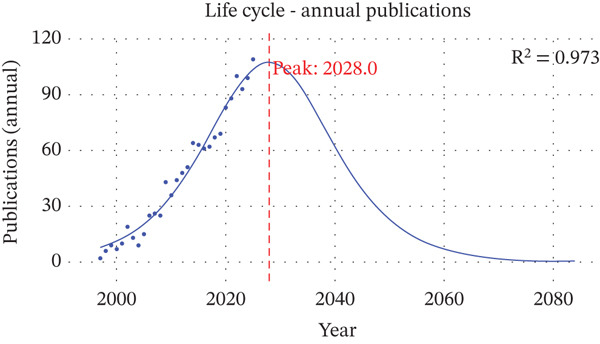
Life cycle model and projected peak of annual publications in this research field.

Annual publication output on clinically translatable variant‐based biomarkers in ascending aortic aneurysm shows a clear multiphase expansion pattern, with both absolute volume and relative contribution to the corpus steadily increasing over nearly three decades (Figure 3, Table 1). In the initial exploratory phase from 1997 to 2004, annual articles remained below 20 and accounted for < 1.5% of all included publications, reflecting early gene discovery work focused on syndromic thoracic aortic disease under conditions of limited sequencing capacity. From 2005 to 2013, a growth phase emerged: Annual output rose from 15 to 51 papers, and the yearly proportion increased to 3.79%, coinciding with the wider availability of next‐generation sequencing, the characterization of key causal genes in Marfan and Loeys–Dietz syndromes, and increasing recognition of heritable thoracic aortic aneurysm and dissection in clinical guidelines, all of which created demand for variant‐informed risk stratification. A consolidation and expansion phase is evident from 2014 onwards, with output stabilizing around 60–70 papers until 2018 and then accelerating again to 83–109 articles per year between 2020 and 2025, during which the annual share nearly doubled from 4.75% to 8.10%. This second surge reflects the diffusion of panel and exome sequencing into routine cardiovascular practice, the establishment of multicenter aortopathy registries, and a shift from isolated case reports toward larger cohorts and genotype–phenotype correlation studies that directly inform surgical timing and family screening. Life cycle modeling of annual publications suggests that, although the field is still in a growth stage, it is approaching a maturity peak around 2028, after which a gradual decline in new publications is projected (Figure 4). This projected plateau implies that discovery of novel high‐penetrance genes may slow and that research will increasingly concentrate on refining variant interpretation, integrating multiomics and imaging biomarkers, and implementing existing variant‐based tools in clinical decision pathways rather than expanding the gene catalog itself.

**Table 1:** Annual publication volume and percentage of total.

Year	Articles	Percentage of the total
1997	2	0.15%
1998	6	0.45%
1999	9	0.67%
2000	7	0.52%
2001	10	0.74%
2002	19	1.41%
2003	13	0.97%
2004	9	0.67%
2005	15	1.11%
2006	25	1.86%
2007	26	1.93%
2008	25	1.86%
2009	43	3.19%
2010	36	2.67%
2011	44	3.27%
2012	48	3.57%
2013	51	3.79%
2014	64	4.75%
2015	63	4.68%
2016	61	4.53%
2017	62	4.61%
2018	67	4.98%
2019	69	5.13%
2020	83	6.17%
2021	88	6.54%
2022	100	7.43%
2023	93	6.91%
2024	99	7.36%
2025	109	8.10%

### 3.3. National Co‐Occurrence Analysis

**Figure 5:**
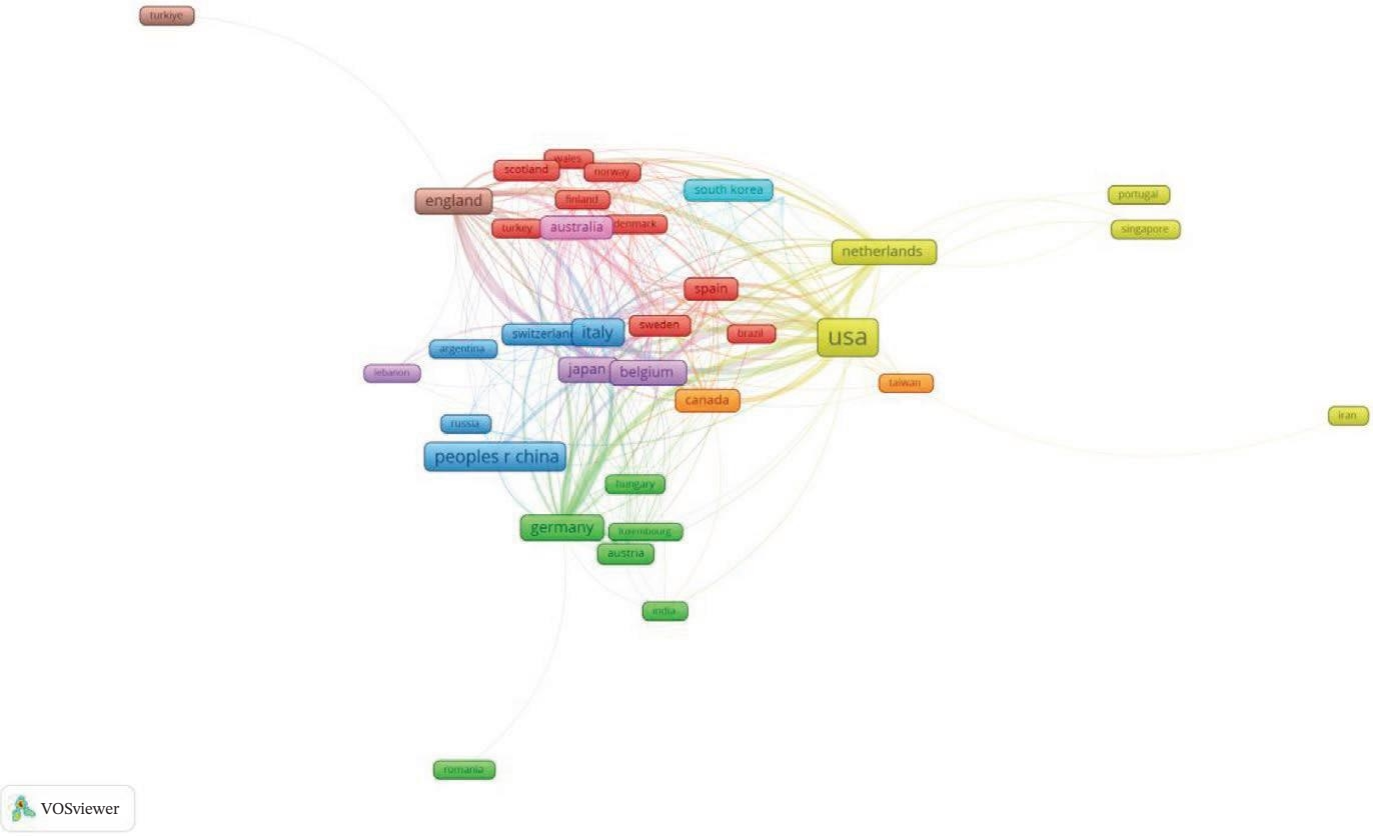
International co‐authorship network of countries publishing on variant‐based biomarkers in ascending aortic aneurysm.

National co‐occurrence analysis shows that research on clinically translatable variant‐based biomarkers in ascending aortic aneurysm is geographically concentrated yet internationally networked, with 50 countries contributing to the literature (Figure 5 and Figures S1 and S2, Table S1). The United States is the dominant hub, producing 405 articles and 17,383 citations and occupying the central position in the collaboration network, with dense co‐authorship links to Western Europe, East Asia, and Oceania. This centrality is consistent with the presence of long‐standing aortic centers, large heritable thoracic aortic disease registries, and early adoption of next‐generation sequencing in cardiovascular genetics, which together generate both high patient volumes and complex variant datasets that require multinational expertise. Western European countries form a tightly connected cluster anchored by Italy, Germany, France, the Netherlands, Belgium, and the United Kingdom, which together contribute a substantial share of output and, in several cases, high citation intensity; Belgium, for example, publishes 47 articles but accrues 4599 citations, reflecting influential methodological and cohort studies that are widely reused by other groups. China is now the second‐largest producer by volume (155 articles) but has a lower citation rate per paper than most Western countries, suggesting a field that is rapidly expanding but still consolidating its global impact, likely due to the more recent establishment of cardiovascular genetics programs and fewer long‐term follow‐up cohorts. Other high‐income countries such as Japan, Canada, Spain, Korea, and Australia contribute moderate output and participate in the main collaboration core, while many middle‐ and low‐income countries appear at the periphery with sparse links and few citations, indicating that technical requirements (advanced imaging, genetic testing, and specialized surgical services) and funding constraints limit their participation. The co‐occurrence patterns depict a field driven by North American and Western European centers, with emerging contributions from East Asia.

### 3.4. Analysis of Collaborative Models Among Countries

Analysis of corresponding author affiliations and cross‐national links indicates that research on clinically translatable variant‐based biomarkers in ascending aortic aneurysm is organized around a small number of high‐output hubs embedded in dense international networks rather than isolated national efforts. In bibliometric terms, corresponding author leadership denotes the country that provides the formal coordinating contact on the publication record rather than exclusive intellectual ownership, which in consortia may be shared across recruiting, phenotyping, sequencing, and analytic teams. The United States is the primary coordinating center, contributing well over 400 corresponding author papers, of which a substantial share are SCPs, indicating strong domestic capacity to recruit patients, perform advanced imaging and sequencing, and complete analyses within national infrastructures (Figure S3). China ranks second in output with a similar predominance of SCPs, reflecting the rapid expansion of cardiovascular genetics programs and large internal patient populations that allow many projects to be conducted without external partners. In contrast, European countries such as Italy, Germany, France, the United Kingdom, the Netherlands, and Belgium show a more balanced or even high proportion of MCPs, consistent with a collaborative model driven by geographically proximate centers, shared EU funding schemes, and long‐standing multinational aortopathy registries that necessitate cross‐border data pooling. The global collaboration map reveals a core‐periphery structure in which the United States, Western Europe, China, and Australia form an interconnected backbone linked by numerous bidirectional partnerships, while institutions in South America, Eastern Europe, the Middle East, and South Asia participate more sporadically and predominantly through collaborations with these hubs (Figure S4). This pattern arises from the combination of a rare but clinically severe disease, which requires aggregation of patients and families across centers to achieve adequate power for variant discovery, and the concentration of high‐throughput sequencing, genetic counseling, and complex aortic surgery facilities in high‐income countries. The dominant collaborative model in this field is a hub‐and‐spoke network of multicenter consortia anchored in North America, Western Europe, and East Asia, through which peripheral countries gain access to genomics platforms and expertise while contributing additional cases that improve the generalizability and clinical translatability of variant‐based biomarkers.

### 3.5. Co‐Occurrence Analysis of Institutions

Institution‐level co‐occurrence analysis reveals a densely interconnected but clearly structured collaboration network in which a limited number of high‐volume academic medical centers act as organizing hubs for research on variant‐based biomarkers in ascending aortic aneurysm (Figures S5–S7). Institutions from France and the United States occupy particularly central positions: Université Paris Cité and Assistance Publique‐Hôpitaux de Paris (AP‐HP) contribute 271 and 261 articles, respectively, while Harvard University, INSERM, the University of Texas System, Johns Hopkins University, Harvard University Medical Affiliates, and the Mayo Clinic each exceed 100 publications, collectively forming a transatlantic core of expertise (Figure S7). In the co‐authorship network, these centers are linked by dense edges to specialized hospitals such as Hôpital Bichat‐Claude Bernard, Ghent University Hospital, Erasmus MC, and multiple North American children′s hospitals, indicating that most studies rely on multi‐institutional cohorts that pool complex surgical cases and family‐based genetic data rather than single‐center series (Figure S5). The temporal overlay map shows that several European institutions (e.g., Hannover Medical School, CHU Dijon, and Erasmus MC) and AP‐HP were early contributors, whereas US centers such as Baylor College of Medicine, the University of Texas Health Science Center at Houston, and Mayo Clinic have sustained or more recent high activity, and Chinese institutions including Capital Medical University and Sun Yat‐sen University emerge as newer nodes entering the network after 2015 (Figure S6). This pattern reflects the clinical and logistical requirements of the field: Ascending aortic aneurysm is relatively rare, but its management demands high‐volume cardiovascular surgery programs, advanced imaging, and access to genome‐scale testing, so only large tertiary centers and national research institutes can systematically accumulate sufficient cases and biospecimens. As a result, the dominant institutional model is that of geographically distributed, hospital‐based consortia anchored in a few leading universities and public hospital systems, which coordinate longitudinal registries and translational projects that transform variant discovery into clinically deployable risk stratification tools.

### 3.6. Journal Analysis

At the journal level, research on clinically translatable variant‐based biomarkers in ascending aortic aneurysm is distributed across a tightly connected set of cardiovascular, surgical, genetics, and rare disease outlets, forming an integrated but clearly multidomain publication ecosystem (Figure S8). The co‐citation network places *American Journal of Medical Genetics Part A* at the center, surrounded by clinical genetics and human genetics journals such as *Genetics in Medicine*, *Clinical Genetics*, *European Journal of Human Genetics*, and *Orphanet Journal of Rare Diseases*, as well as cardiovascular and surgical journals including *Journal of Thoracic and Cardiovascular Surgery*, *Journal of Vascular Surgery*, *International Journal of Cardiology*, and *Frontiers in Cardiovascular Medicine*, indicating that translational work typically couples variant discovery with detailed phenotyping and management data across specialties. Frequency analysis confirms this pattern: *American Journal of Medical Genetics Part A* publishes the largest number of articles (*n* = 60), followed by *Genetics in Medicine* and *Journal of Thoracic and Cardiovascular Surgery* (each *n* = 25), *Journal of Vascular Surgery* (*n* = 24), and *European Journal of Human Genetics* and *Frontiers in Cardiovascular Medicine* (each *n* = 22), reflecting a balance between genetics‐focused and cardiovascular surgery–focused venues (Figure S9). In contrast, the most locally cited sources are dominated by high‐impact general cardiology and human genetics journals; *Circulation* and *Nature Genetics* together account for nearly 4500 citations, ahead of *Journal of the American College of Cardiology*, *American Journal of Human Genetics*, and *New England Journal of Medicine* (Figure S10), showing that foundational evidence on thoracic aortic aneurysm pathophysiology and key gene discoveries is often published in broad‐scope journals and subsequently cited by more specialized outlets. The temporal overlay in Figure S8 suggests an evolution from earlier reliance on traditional cardiology and vascular surgery journals (blue nodes) toward increasing use of genetics and multidisciplinary platforms, including newer open‐access titles (greener/yellower nodes), paralleling the shift from isolated case series toward genome‐wide approaches and functional studies. These patterns indicate that the field is anchored in specialty genetics and cardiovascular journals for routine dissemination, while conceptually and methodologically, it depends on discoveries and guidelines originating from a small set of high‐impact cardiology and human genetics sources.

### 3.7. Keyword Co‐Occurrence Analysis

Keyword co‐occurrence mapping shows that research on clinically translatable variant‐based biomarkers in ascending aortic aneurysm is organized around a central axis linking “mutations” with syndromic entities and clinical outcomes (Figure S11). The most frequent terms were “mutations” (*n* = 378), “marfan syndrome”/“marfan‐syndrome” (*n* = 277 and 269), “aneurysm” (*n* = 183), “diagnosis” (*n* = 159), “dissection” (*n* = 155), “management” (*n* = 146), “disease” (*n* = 144), and “loeys‐dietz syndrome” (*n* = 134), indicating that the core intellectual focus is on genetically mediated thoracic aortopathies and their translation into diagnostic and therapeutic decision‐making (Figure S13). In the network, the first major cluster couples “marfan syndrome,” “loeys‐dietz syndrome,” “ehlers‐danlos syndrome,” “fbn1,” “tgfbr1/2,” and “genotype‐phenotype correlation,” representing work that defines causal variants, inheritance patterns, and reproducible links between specific genes or variant classes and observed risk profiles in heritable thoracic aortic disease. A second cluster centers on “aneurysm,” “aortic dissection,” “ascending aorta,” “bicuspid aortic valve,” and “aortopathy,” reflecting efforts to integrate genetic information with imaging‐based assessment of aortic size progression and acute complications. A third mechanism‐oriented cluster groups “tgf‐beta,” “fibrillin‐1,” “extracellular matrix,” “smooth‐muscle cells,” and “pathogenesis,” highlighting ongoing interest in signaling and structural pathways that may yield mechanistically grounded biomarkers or targeted therapies. Terms related to “diagnosis,” “genetic testing,” “guidelines,” “management,” “surgery,” “replacement,” “risk,” and “pregnancy” connect these clusters and point to a sustained emphasis on implementing variant findings in clinical practice, including timing of prophylactic surgery and management of high‐risk life events. The temporal overlay suggests an evolution from earlier basic and syndrome‐defining work (cooler colors) toward more recent topics, such as registries, guideline development, and risk stratification (warmer colors), while the density map confirms that the highest conceptual intensity lies at the interface of “mutations,” “marfan syndrome,” “aneurysm,” “dissection,” and “diagnosis” (Figure S12). Overall, the keyword structure reflects a field driven by the need to move from gene discovery in rare heritable aortopathies toward clinically actionable variant‐based tools that refine diagnosis, prognosis, and management of ascending aortic aneurysm.

### 3.8. Trend Topic Analysis

Trend topic analysis shows a clear thematic progression from gene discovery and basic characterization toward integrated management of heritable thoracic aortic disease (Figure S14). The earliest high‐frequency terms, appearing around 2000–2005, cluster around structural biology and pedigree‐based genetics, including “fibrillin gene,” “fibrillin‐1,” “EGF‐like domain,” “linkage analysis,” “familial abdominal aortic aneurysm,” and “disorder,” together with imaging‐related terms such as “transesophageal echocardiography” and “intracranial aneurysm,” reflecting an initial phase focused on identifying FBN1 variants, describing connective tissue manifestations, and defining the vascular phenotype in Marfan families. Between 2005 and 2012, topics shifted toward mechanistic and syndromic refinement, with sustained prominence of “FBN1 mutation,” “mouse model,” “pathogenesis,” “TGF‐beta,” “thoracic aortic‐aneurysm,” and “nosology,” indicating efforts to map TGF‐beta signaling pathways, develop animal models, and formalize diagnostic criteria for Marfan and related syndromes. From approximately 2012 onwards, the dominant terms became more clinically oriented: “mutations,” “aneurysm,” “aortic dissection,” “diagnosis,” “management,” “cardiovascular disease,” and “life expectancy,” consistent with the diffusion of next‐generation sequencing into practice and the need to translate expanding variant catalogs into risk stratification and surgical decision‐making. The most recent emerging topics, concentrated after 2016, include “heritable thoracic aortic disease,” “thoracic aortic dissection,” “vascular Ehlers‐Danlos syndrome,” “classification,” “variants,” “genetic testing,” “guidelines,” “association,” and “Mendelian randomization,” marking a transition toward umbrella concepts that group multiple syndromes, systematic variant interpretation frameworks, formal guideline development, and causal inference approaches that link specific variants or pathways to outcomes. This temporal layering suggests that the field has progressed from describing single‐gene Marfan pathology to a broader concept of heritable thoracic aortic disease in which multigene panels, standardized classification systems, and population‐based evidence are used to refine variant‐based biomarkers and embed them in clinical algorithms for ascending aortic aneurysm care.

## 4. Discussion

This cross‐database bibliometric analysis of 1346 publications on clinically translatable variant‐based biomarkers in ascending aortic aneurysm delineates a dynamic yet progressively consolidating research domain spanning nearly three decades. We observed a sustained rise in annual output from sporadic case‐based reports in the late 1990s to more than 100 articles per year by 2025, accompanied by a relatively high average citation count and dense co‐authorship networks. Together, these features indicate that variant‐based biomarker research in ascending aortic aneurysm has moved from peripheral curiosity toward an increasingly mature, collaboration‐intensive field, in which foundational gene discovery work continues to exert substantial influence, while newer translational studies incrementally reshape clinical practice [27–29]. At the same time, life cycle modeling suggests that the field is approaching a publication peak, implying a gradual shift away from the expansive identification of novel high‐penetrance genes toward refinement [30, 31], implementation, and integration of existing variant‐based tools into risk stratification, surveillance, and decision‐making pathways for heritable thoracic aortic disease.

The temporal trajectory and thematic layering we observed are broadly concordant with the evolution of heritable thoracic aortic disease research more generally, yet they also highlight distinct features of the variant‐focused translational literature. Read narratively, the field progressed through four overlapping phases: early gene discovery, panel‐era consolidation, large‐scale sequencing and registry‐driven consortia, and the current emphasis on precision‐risk and management questions (Table 2). Early trend topics centered on fibrillin‐1, EGF‐like domains, linkage analysis, and pedigree‐based descriptions of Marfan families, reflecting the period in which FBN1‐linked disease was cemented as a causal substrate for ascending aortic aneurysm and dissection [21, 22]. The next phase broadened the architecture of heritable aortopathy through TGF‐beta signaling, mouse models, refined nosology, and panel‐based testing, while the subsequent consortium/sequencing phase expanded cohort size, long‐term follow‐up, and reproducible genotype–phenotype analyses [23, 32–35]. The most recent literature is increasingly organized around precision‐risk and management questions: variant interpretation, genotype‐specific operative thresholds, pregnancy and life‐course counseling, implementation of family screening, and integration of imaging or functional evidence into clinical decisions. This evolution underscores that variant‐based biomarkers have served not only as mechanistic clues but also as anchors for redefining disease entities, risk thresholds, and life‐course management strategies.

**Table 2:** Clinician‐oriented summary of major scientific advances and milestones across developmental phases of the field.

Developmental phase	Major scientific advances and milestones	Main translational consequence
Early gene discovery (1997–2004)	Pedigree analysis and syndrome definition studies, FBN1‐centered aortopathy work, and recognition that heritable variants can underlie ascending aneurysm and dissection	Established monogenic aortopathy as a clinically relevant substrate for diagnosis and family screening
Panel‐era consolidation (2005–2013)	Expansion to TGF‐beta pathway genes and related syndromes, refined nosology, and wider use of targeted gene panels	Broadened genetic diagnosis and early genotype–phenotype risk stratification
Large‐scale sequencing and consortia (2014–2018)	NGS/exome adoption, multicenter registries, curated gene‐validity frameworks, and larger cohort and follow‐up studies	Shifted the evidence base from isolated cases to reproducible cohort‐based estimates of growth, dissection, and family risk
Precision‐risk and management implementation (2019–2025)	Variant curation, guideline integration, radiogenomic/multiomic expansion, and pragmatic decision‐support tools	Focused the field on VUS handling, ancestry diversity, genotype‐guided operative thresholds, pregnancy/life‐course counseling, and real‐world implementation

Our country‐level and institutional co‐authorship analyses reveal a geographically concentrated yet highly networked architecture in which a small number of high‐income countries and tertiary academic centers act as structural hubs. The United States, together with Western European countries such as Italy, Germany, France, the Netherlands, Belgium, and the United Kingdom, occupies the densest region of the collaboration map and accumulates the majority of citations. This prominence likely reflects a confluence of factors: long‐standing aortic reference centers, early adoption of next‐generation sequencing in cardiovascular genetics, the presence of multigenerational registries and large family‐based cohorts, and access to complex aortic surgery, imaging, and multidisciplinary clinics that can systematically integrate genotype–phenotype information into care pathways [36–38]. By contrast, China has rapidly emerged as the second largest contributor by publication volume but with lower average citation impact, consistent with a field that is expanding in scale yet still developing its global influence, an evolution observed previously in other cardiovascular and genomic domains. Countries from South America, Eastern Europe, the Middle East, South Asia, and Africa tend to occupy the periphery of the network with sparse connections and low citation counts, highlighting enduring inequities in access to genomic technologies, specialized surgical care, and cross‐border consortia [39, 40]. For rare but highly penetrant conditions such as heritable ascending aortic aneurysm, these structural disparities risk entrenching a scenario in which most variant‐based biomarkers and associated management thresholds are derived from and validated in relatively homogeneous populations, with uncertain generalizability to underrepresented regions and ancestries.

At the institutional level, our findings emphasize the centrality of large university hospitals and national research institutes in driving clinically relevant variant‐based biomarker work. French institutions (Université Paris Cité and AP‐HP) and major North American centers (Harvard University and affiliates, INSERM, the University of Texas System, Johns Hopkins University, and Mayo Clinic) form a transatlantic backbone that coordinates multicenter registries and shared biobanks. The dense interconnections between these hubs and specialized surgical and pediatric centers reflect the practical realities of this research area: to obtain sufficient numbers of genotyped patients with detailed imaging, longitudinal follow‐up, and surgical outcomes, investigators must pool cases across institutions and, increasingly, across countries. The temporal overlays suggest that some European centers were early adopters, with later waves of high activity in North America and, more recently, growing contributions from Chinese institutions such as Capital Medical University and Sun Yat‐sen University. This pattern supports an interpretation in which variant‐based aortopathy research spreads outward from early pioneer centers to newer programs that leverage falling sequencing costs, expanding national insurance coverage for genetic testing, and heightened guideline emphasis on family screening.

The journal ecosystem mapped in this study further illustrates how variant‐based biomarker research in ascending aortic aneurysm sits at the interface of human genetics, cardiovascular medicine, and surgery. The fact that *American Journal of Medical Genetics Part A*, *Genetics in Medicine*, and *European Journal of Human Genetics* appear alongside *Journal of Thoracic and Cardiovascular Surgery*, *Journal of Vascular Surgery*, and dedicated cardiovascular medicine journals among the most productive outlets indicates that clinically translatable work typically entails both rigorous genetic characterization and detailed phenotyping of aortic dimensions, valve morphology, dissection events, and operative outcomes. At the same time, the concentration of local citations in broad‐scope, high‐impact journals such as *Circulation*, *Nature Genetics*, *Journal of the American College of Cardiology*, *American Journal of Human Genetics*, and *New England Journal of Medicine* underscores that many of the field′s formative discoveries, including identification of key genes, pathophysiological pathways, and risk thresholds, were initially disseminated through general cardiovascular or genetics platforms before being elaborated in more specialized venues. The temporal shift from earlier reliance on traditional cardiology and vascular surgery outlets toward increasing use of genetics and multidisciplinary journals, including newer open‐access titles, aligns with the field′s transition from isolated case series and descriptive reports to panel‐based testing, variant reclassification, guideline development, and integrative translational studies.

Keyword co‐occurrence and trend topic analyses offer additional insight into the conceptual backbone of this literature and where translational opportunities remain. The central axis connecting “mutations,” “marfan syndrome,” “loeys‐dietz syndrome,” “aneurysm,” “aortic dissection,” “diagnosis,” and “management” indicates that for much of the field′s history, variant‐based biomarkers have been developed within the framework of rare high‐penetrance syndromic conditions [41–43]. Mechanism‐oriented terms related to TGF‐beta signaling, extracellular matrix biology, and smooth muscle cell dysfunction are tightly coupled to these syndromes, reflecting an enduring focus on pathways that might yield therapeutic targets as well as diagnostic markers [44, 45]. More recent emergence of umbrella concepts, such as “heritable thoracic aortic disease,” alongside “variants,” “genetic testing,” “guidelines,” and “classification,” suggests a broadening toward integrated risk frameworks that span multiple genes and clinical phenotypes and explicitly link variant profiles to surgical indications and surveillance strategies [46–48]. At the same time, the bibliometric distribution implies that attention has been disproportionately drawn to classical syndromic high‐penetrance genes and pathway biology, whereas several clinically important areas remain comparatively thin: structured management of variants of uncertain significance, bicuspid‐versus‐familial overlap phenotypes, ancestry‐specific interpretation, prospective evidence on genotype‐guided operative thresholds, and integration of polygenic, multiomic, or imaging‐derived markers alongside monogenic variants [49, 50]. This gap reinforces the notion that the field is transitioning from discovery to implementation but has not yet fully capitalized on the convergence of high‐throughput functional assays, machine learning, and diverse population‐based cohorts.

Several strengths of our study support the validity of these observations. First, we drew on three major databases (Web of Science Core Collection, Scopus, and PubMed) and implemented a structured harmonization, deduplication, and field standardization pipeline, thereby minimizing single‐database bias and improving the completeness of citation and keyword information. Second, the concept‐driven search strategy explicitly integrated ascending aortic aneurysm phenotypes, genomic variation, and clinically oriented biomarker/translation terms, increasing the likelihood that the corpus captured studies where variant data were meaningfully connected to diagnosis, prognosis, or management rather than appearing as incidental genetic observations. Third, we combined complementary science mapping tools and network perspectives, including co‐authorship, co‐citation, bibliographic coupling, keyword co‐occurrence, collaboration maps, and trend topics, under a consistent thesaurus and counting framework, which allowed convergent triangulation on the field′s structural and thematic cores. Finally, we summarized both productivity and impact metrics at multiple levels (journals, authors, institutions, and countries), thereby avoiding overreliance on simple counts that might conflate volume with influence. Nonetheless, several limitations should be acknowledged when interpreting our findings. As with all bibliometric analyses, our results are constrained by the coverage, indexing practices, and citation dynamics of the underlying databases. Despite cross‐database integration, relevant studies indexed only in regional or specialty databases or those published in nonindexed local journals may have been missed, potentially underestimating contributions from low‐ and middle‐income countries. Our reliance on titles, abstracts, and indexed terms for screening and thematic mapping means that misclassification of scope or topic is possible, particularly for older records with sparse metadata. Citation counts were treated as a snapshot anchored to the extraction date and do not capture the future impact of recent publications; highly cited older papers may therefore dominate co‐citation networks even as newer work begins to reshape guidelines and practice [24–26]. More broadly, citation‐based impact should not be interpreted as a pure measure of scientific quality because it is also shaped by database coverage, English‐language visibility, publication age, and field‐specific citation behavior [24–26]. Language bias cannot be excluded, as English‐language journals predominate in the major databases, and non‐English work may be underrepresented. Although we applied rule‐based cleaning and targeted disambiguation for authors, institutions, and keywords, residual fragmentation or conflation of entities is likely, especially for common surnames and evolving institutional names; likewise, even carefully specified merge and nonmerge rules can compress nuance between closely related phenotypes or variant classes. Finally, bibliometric and science mapping methods can describe publication structures and intellectual linkages but cannot directly assess the clinical validity, utility, or equity of specific variant‐based biomarkers; these require complementary systematic reviews, variant curation efforts, and prospective outcome studies.

The dominance of a relatively small group of countries and institutions suggests that broadening participation through support for emerging centers, deliberate inclusion of diverse ancestries, and equitable access to genomic technologies will be essential to ensure that variant‐based risk thresholds, surveillance intervals, and surgical indications are generalizable rather than tailored to a narrow subset of patients [51, 52]. The journal and keyword landscapes highlight a maturing translational pipeline in which monogenic high‐penetrance variants form the backbone of heritable thoracic aortic disease management, while emerging themes point toward integration of multigene panels, functional assays, and causal inference approaches such as Mendelian randomization. Scientifically and practically, the next priorities are to link genotype to hard outcomes in prospective registries, standardize VUS adjudication and recontact workflows, expand representation of diverse ancestries and low‐resource settings, and test when genetic results actually change imaging intervals, pregnancy counseling, or operative timing in routine practice. Funders and research networks might use these findings to identify underserved topics (e.g., long‐term real‐world outcomes of genotype‐guided surgery, systematic handling of variants of uncertain significance, and incorporation of ancestry‐specific modifiers) and to design multicenter consortia that bridge currently disconnected clusters. For guideline developers and clinicians, recognizing where evidence is dense versus sparse can help prioritize areas in which variant‐based biomarkers are ready for routine deployment and those where further analytic validation and clinical utility studies are needed. Future work that couples updated bibliometric tracking with curated variant databases, registry‐based outcome studies, pragmatic implementation science, and economic evaluations will be critical to transform the rich but uneven variant‐based biomarker landscape into equitable, clinically robust tools for preventing dissection and improving survival in patients with ascending aortic aneurysm.

## 5. Conclusion

This study shows that, although research on clinically translatable variant‐based biomarkers in ascending aortic aneurysm has expanded into a mature, collaboration‐intensive domain, its thematic focus and global participation remain uneven, with discovery efforts still outpacing rigorous clinical implementation. Citation density and network centrality alone do not guarantee patient benefit, yet the tightly knit journal, country, and institutional cores we identified, organized around FBN1‐related disease, TGF‐beta pathway syndromes, and heritable thoracic aortic disease, foreshadow an era in which variant‐informed classification, surveillance, and surgical timing become embedded in routine care. The persistent gap between high‐output hubs and underrepresented regions and between monogenic high‐penetrance markers and emerging polygenic, multiomic, and imaging‐based signals highlights how concentrated infrastructure and long‐standing registries sharpen biomarker translation, whereas settings with limited resources remain vulnerable to extrapolation from narrowly sampled populations. Even so, nuanced genotype–phenotype correlation, ancestry‐conscious variant interpretation, harmonized VUS curation, and prospective outcome validation, particularly in diverse healthcare systems and family‐based registries, remain indispensable to ensure that variant‐based tools refine rather than rigidify risk thresholds for dissection prevention. Future work should focus on multinational, multilevel consortia that weld established gene‐based frameworks to curated variant databases, longitudinal registries, and implementation science, thereby advancing earlier diagnosis, more equitable risk stratification, and truly clinically translatable variant‐based biomarkers for ascending aortic aneurysm worldwide.

## Author Contributions

Conceptualization: Fan Yang, Tan Yang, Xiaojun Xie, Qi Yang, and Fang Wang. Methodology: Fan Yang, Tan Yang, Xiaojun Xie, Qi Yang, and Fang Wang. Validation: Fan Yang, Tan Yang, Xiaojun Xie, Qi Yang, and Fang Wang. Formal analysis: Fan Yang, Tan Yang, Xiaojun Xie, Qi Yang, and Fang Wang. Investigation: Fan Yang, Tan Yang, Xiaojun Xie, Qi Yang, and Fang Wang. Resources: Fan Yang, Tan Yang, Xiaojun Xie, Qi Yang, and Fang Wang. Data Curation: Fan Yang, Tan Yang, Xiaojun Xie, Qi Yang, and Fang Wang. Writing—original draft: Fan Yang, Tan Yang, Xiaojun Xie, Qi Yang, and Fang Wang. Writing—review and editing: Fan Yang, Tan Yang, Xiaojun Xie, Qi Yang, and Fang Wang. Project administration: Fan Yang, Tan Yang, Xiaojun Xie, Qi Yang, and Fang Wang.

## Funding

This research was supported by the University‐level Project 2023QN009 of Southwest Medical University.

## Ethics Statement

This study has been reviewed and approved by the Ethics Committee of Southwest Medical University, which has determined that it constitutes a bibliometric research project. As it does not involve any human or animal experimentation, ethical approval is not required for its conduct.

## Conflicts of Interest

The authors declare no conflicts of interest.

## Supporting information


**Supporting Information** Additional supporting information can be found online in the Supporting Information section. Figures S1, S2, S3, S4, S5, S6, S7, S8, S9, S10, S11, S12, S13, and S14 and Table S1 are provided as separate supporting information files. Figures S1 and S2 and Table S1: Summary of country‐level production, citation, and geographic distribution patterns. Figures S3 and S4: Single‐versus multiple‐country publication patterns and global collaboration links. Figures S5–S7: Detail of institutional collaboration networks and productive institutions. Figures S8–S10: A summary of journal co‐citation/publication networks and locally cited journals. Figures S11–S14: Keyword co‐occurrence, thematic density, frequent‐keyword, and trend topic analyses. These supporting information materials support the national, institutional, journal, keyword, and trend topic analyses reported in Sections 3.3–3.8.

## Data Availability

The data that support the findings of this study are available from the corresponding author upon reasonable request.
