# Supplementary material for: Clinically Translatable Mutation‐Based Biomarkers in Ascending Aortic Aneurysm: A Bibliometric Study
Source: Hum Mutat. 2026 May 25;2026:3155191. doi: 10.1155/humu/3155191 (PMC13200172; doi:10.1155/humu/3155191)
Supplement: Supplementary file 1 — Supporting Information Additional supporting information can be found online in the Supporting Information section. Figures S1, S2, S3, S4, S5, S6, S7, S8, S9, S10, S11, S12, S13, and S14 and Table S1 are provided as separate supporting information files. Figures S1 and S2 and Table S1: Summary of country‐level production, citation, and geographic distribution patterns. Figures S3 and S4: Single‐versus multiple‐country publication patterns and global collaboration links. Figures S5–S7: Detail of institutional collaboration networks and productive institutions. Figures S8–S10: A summary of journal co‐citation/publication networks and locally cited journals. Figures S11–S14: Keyword co‐occurrence, thematic density, frequent‐keyword, and trend topic analyses. These supporting information materials support the national, institutional, journal, keyword, and trend topic analyses reported in Sections 3.3–3.8. [file HUMU-2026-3155191-s001.zip › Supplementary Table 1.docx]

**Supplementary Table 1. Publication Volume and Citation Counts by Country.**

| **Country** | **Articles** | **Total Citations** |
| --- | --- | --- |
| USA | 405 | 17383 |
| CHINA | 155 | 1681 |
| ITALY | 99 | 1946 |
| GERMANY | 80 | 1964 |
| FRANCE | 78 | 3264 |
| UNITED KINGDOM | 71 | 1248 |
| NETHERLANDS | 60 | 2580 |
| JAPAN | 50 | 700 |
| BELGIUM | 47 | 4599 |
| CANADA | 41 | 880 |
| SPAIN | 40 | 985 |
| KOREA | 29 | 581 |
| AUSTRALIA | 20 | 212 |
| POLAND | 17 | 190 |
| TURKEY | 11 | 204 |
| NORWAY | 10 | 158 |
| SWITZERLAND | 10 | 227 |
| AUSTRIA | 8 | 212 |
| INDIA | 8 | 51 |
| MEXICO | 8 | 60 |
| ROMANIA | 8 | 26 |
| HUNGARY | 6 | 70 |
| IRAN | 5 | 18 |
| PORTUGAL | 5 | 70 |
| RUSSIA | 5 | 103 |
| SWEDEN | 5 | 85 |
| DENMARK | 4 | 181 |
| ISRAEL | 4 | 138 |
| LITHUANIA | 4 | 23 |
| SINGAPORE | 4 | 72 |
| ARGENTINA | 3 | 47 |
| BRAZIL | 3 | 31 |
| CZECH REPUBLIC | 3 | 29 |
| FINLAND | 3 | 593 |
| GREECE | 3 | 29 |
| COLOMBIA | 2 | 3 |
| CYPRUS | 2 | 11 |
| EGYPT | 2 | 21 |
| SLOVENIA | 2 | 14 |
| THAILAND | 2 | 6 |
| UKRAINE | 2 | 5 |
| BAHRAIN | 1 | 54 |
| CHILE | 1 | 1 |
| ICELAND | 1 | 7 |
| IRELAND | 1 | 1 |
| LEBANON | 1 | 0 |
| LUXEMBOURG | 1 | 21 |
| SAUDI ARABIA | 1 | 1 |
| SOUTH AFRICA | 1 | 4 |
| U ARAB EMIRATES | 1 | 0 |
