# Supplementary figures and images for: Clinically Translatable Mutation‐Based Biomarkers in Ascending Aortic Aneurysm: A Bibliometric Study
Source: Hum Mutat. 2026 May 25;2026:3155191. doi: 10.1155/humu/3155191 (PMC13200172; doi:10.1155/humu/3155191)

**blommal Kets in ascending aortic aneurysm.**

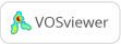

Supplement: Supplementary file 1 — Supporting Information Additional supporting information can be found online in the Supporting Information section. Figures S1, S2, S3, S4, S5, S6, S7, S8, S9, S10, S11, S12, S13, and S14 and Table S1 are provided as separate supporting information files. Figures S1 and S2 and Table S1: Summary of country‐level production, citation, and geographic distribution patterns. Figures S3 and S4: Single‐versus multiple‐country publication patterns and global collaboration links. Figures S5–S7: Detail of institutional collaboration networks and productive institutions. Figures S8–S10: A summary of journal co‐citation/publication networks and locally cited journals. Figures S11–S14: Keyword co‐occurrence, thematic density, frequent‐keyword, and trend topic analyses. These supporting information materials support the national, institutional, journal, keyword, and trend topic analyses reported in Sections 3.3–3.8. [file HUMU-2026-3155191-s001.zip › Supplementary Figure 11.pdf]

**Supplementary Figure 4. Global collaboration map showing inter-country co-authorship links.**

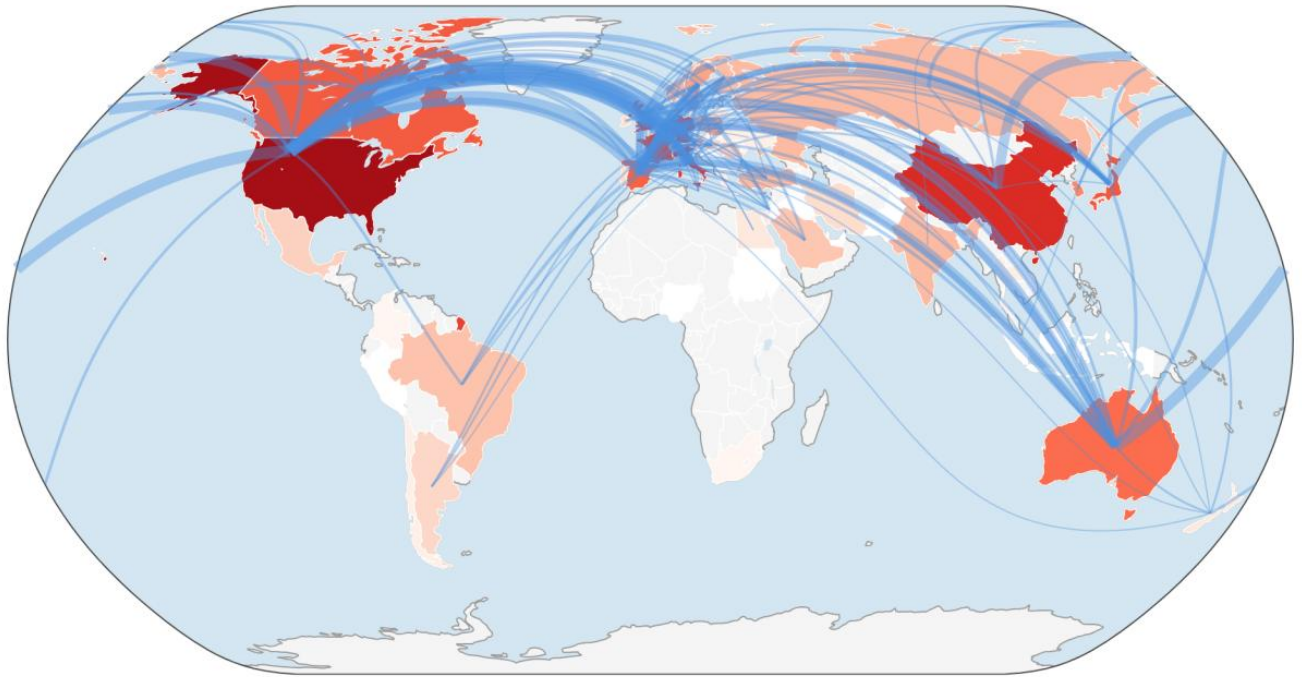

Supplement: Supplementary file 1 — Supporting Information Additional supporting information can be found online in the Supporting Information section. Figures S1, S2, S3, S4, S5, S6, S7, S8, S9, S10, S11, S12, S13, and S14 and Table S1 are provided as separate supporting information files. Figures S1 and S2 and Table S1: Summary of country‐level production, citation, and geographic distribution patterns. Figures S3 and S4: Single‐versus multiple‐country publication patterns and global collaboration links. Figures S5–S7: Detail of institutional collaboration networks and productive institutions. Figures S8–S10: A summary of journal co‐citation/publication networks and locally cited journals. Figures S11–S14: Keyword co‐occurrence, thematic density, frequent‐keyword, and trend topic analyses. These supporting information materials support the national, institutional, journal, keyword, and trend topic analyses reported in Sections 3.3–3.8. [file HUMU-2026-3155191-s001.zip › Supplementary Figure 4.pdf]
